# Deep-Learning Model for Iris and Eyebrow Segmentation and Automation of Eye Landmark Measurements in Acquired Ptosis

**DOI:** 10.3390/biomedicines13102557

**Published:** 2025-10-20

**Authors:** Dain Yoo, Hyun Jin Shin

**Affiliations:** 1 School of Medicine, Konkuk University, Chungju 27478, Republic of Korea; uhooyhoo@kku.ac.kr; 2 Department of Ophthalmology, Konkuk University Medical Center, Seoul 05030, Republic of Korea; 3 Research Institute of Medical Science, Konkuk University, Seoul 05029, Republic of Korea

**Keywords:** deep learning, image segmentation, eyebrows, iris, ptosis

## Abstract

**Background**: Acquired ptosis is a common eyelid disorder in elderly patients, causing visual disturbance and cosmetic concerns. Accurate evaluation of periocular anatomy, including eyebrow and iris position, is essential for surgical planning, but current manual assessments are time-consuming and subjective. **Objectives**: This study aimed to develop deep-learning models for iris and eyebrow segmentation to automate eye landmark measurements and enable objective, standardized analysis in patients with acquired ptosis. **Methods**: We retrospectively collected 612 facial images from 209 ptosis patients. Images were labeled for iris and eyebrow segmentation and split into training, validation, and test sets (8:1:1). A deep-learning model was developed to automatically segment the iris and eyebrow regions and automatically measure seven landmarks: MRD1, MRD2, medial eyebrow end, medial limbus, pupil center, lateral limbus, and lateral eyebrow end. **Results**: The iris segmentation model achieved accuracy of 99.7%, precision of 97.6%, recall of 98.3%, an F1 score of 97.9%, and intersection over union of 95.9%. The corresponding metrics for the eyebrow segmentation model were 98.6%, 92.6%, 91.5%, 91.5%, and 85.0%. The mean absolute percentage error and root mean square error for the automated landmark measurements were 4.00% and 2.48 mm, respectively. **Conclusions**: The high performance of the segmentation models and the automated measurements supports their potential use for objective and standardized analyses of acquired ptosis. These findings may aid the future development of predictive tools for use in surgical planning.

## 1. Introduction

The positions of the eyebrows are essential for expressing emotions and nonverbal communication, and they significantly impact the overall facial appearance [1]. The muscles around the eyes weaken with aging, leading to upper eyelid drooping (ptosis). Patients with ptosis compensate for this by overusing the frontalis muscle, causing the eyebrows to elevate in an attempt to improve their field of vision [2]. Studies indicate that the eyebrow can move upward or downward after ptosis correction surgery [3,4]. In some cases, the eyebrow position will change unexpectedly postsurgery, possibly reducing patient satisfaction. Therefore, accurately predicting and discussing the postoperative eyebrow position with the patient is an important aspect in ptosis surgery planning and for ensuring overall patient satisfaction [5]. Objective quantification is difficult because photographic and manual methods require human choices of baseline, landmarks, and scale, which vary across raters and sessions and reduce reproducibility.

Accurate prediction requires reliable localization of periocular landmarks such as iris and eyebrow. Particularly, the iris provides a stable reference for standardizing distances and alignment across images, improving the comparability of measurements. [6]. However, manual annotation of these features by researchers is not only time- and labor-intensive but also introduces subjectivity, since there are no standardized methods for defining the eyebrow boundaries [7]. This subjectivity leads to inter- and intra-grader variabilities that reduce measurement consistency.

To overcome these limitations, recent advances in artificial intelligence (AI) have been used to automate analysis of facial anatomy and outcome prediction in facial surgery [8,9]. Deep-learning-based image segmentation has become a cornerstone technique in medical imaging. Architectures such as Attention U-Net, TransUNet, or Swin-Transformer-based networks and their variants have achieved high performance in medical image analysis [10,11,12]. Similarly, eyebrow segmentation on public datasets such as HELEN and CelebA demonstrates feasibility in general populations. However, these datasets are primarily composed of facial images of younger Caucasian individuals and do not reflect elderly patients with ptosis, where eyelid drooping and compensatory eyebrow elevation alter periocular anatomy. This background underscores the need for developing models tailored to specific clinical conditions.

Accordingly, we assembled a dataset of 612 image samples from 209 East Asian patients with acquired ptosis and developed a U-Net-based framework to segment the iris and eyebrow and to automated periocular landmark measurements. We aimed to evaluate accuracy, reproducibility, efficiency, and model inference latency, which demonstrates its potential for objective and standardized assessments in clinical settings.

## 2. Materials and Methods

This retrospective in silico cohort study was conducted at the Department of Ophthalmology, Konkuk University Medical Center, Seoul, South Korea. This study adhered to the principles of the Declaration of Helsinki and received approval from the institutional review board and ethics committee of Konkuk University Medical Center (approval number: 2024-05-071).

This study initially enrolled facial images of 459 patients who underwent levator resection for the treatment of bilateral or unilateral involutional ptosis at Konkuk University Hospital between January 2006 and March 2024.

### 2.1. Data Preprocessing and Labeling

Applying the following exclusion criteria resulted in facial images from 243 patients being excluded: (1) low-resolution images, (2) absence of either pre- or postoperative facial images, (3) other types of ptosis (e.g., congenital ptosis or third cranial nerve palsy), (4) conditions affecting the eyebrow position (e.g., facial palsy), or (5) a history of procedures influencing the eyebrow position (e.g., eyebrow lifting or forehead botulinum injection). Applying these criteria, facial images from 216 patients were collected.

Using MediaPipe, an open-source AI-based model developed by Google Research (Mountain View, CA, USA), we detected 478 facial landmarks to crop bilateral facial regions around the eyebrows and iris [13]. Facial images from seven patients were excluded during MediaPipe preprocessing due to recognition issues, leaving 209 patients. MediaPipe consists of three models for facial landmark detection, and a valid facial image must include recognizable key facial structures. However, these seven facial images were captured at a distance that was too close, which resulted in important facial structures such as the mouth or nose being omitted.

From each cropped image, we obtained left and right periorbital images. To maintain shape consistency, the right periorbital images samples were horizontally flipped to align with the left periorbital images.

**Figure 1:**
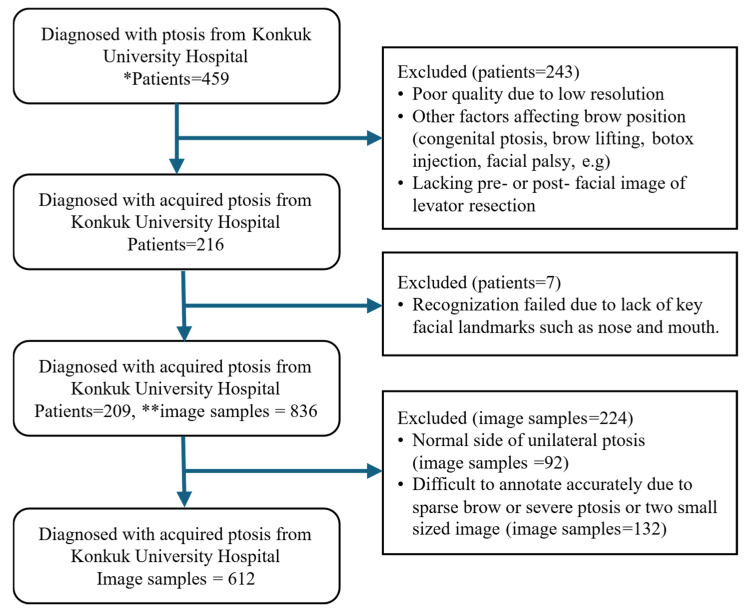
Flow chart of the data exclusion criteria. * Patients: number of individuals who visited Konkuk University Hospital due to acquired ptosis. ** Image samples: number of unilateral periorbital images. Ideally, we obtained four image samples from each patient: two pre- and postoperative facial images, and two hemifacial periorbital images from each facial image. However, in some cases only two image samples were collected from a patient due to unilateral ptosis.

We further excluded 92 image samples that showed no ptosis and 132 images with sparse eyebrows or severe ptosis that made iris and eyebrow identification difficult (Figure 1). This structured approach ensured consistent and accurate segmentation during the model development process [14]. In total, we labeled 612 image samples using the Sefexa tool (http://www.fexovi.com/sefexa.html, accessed on 1 October 2024), an image segmentation tool, with the following annotations:

- Iris segmentation: (1) iris and (2) background.
- Eyebrow segmentation: (1) eyebrow and (2) background.

### 2.2. Data Postprocessing

**Figure 2:**
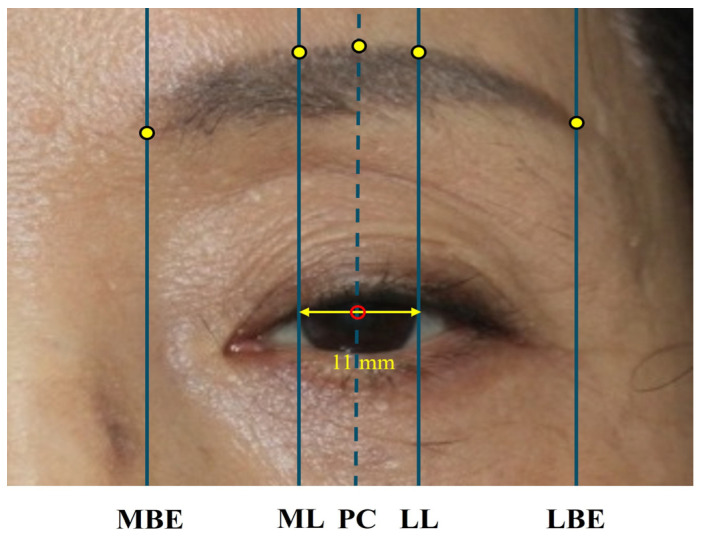
Example of eye landmark measurements. The red circle indicates the pupil center (PC). Using the corneal diameter as a scale bar and the PC as the baseline, distances to the highest point of the eyebrow (yellow dots) were measured at five landmarks: medial eyebrow end (MBE), medial limbus (ML), PC, lateral limbus (LL), and lateral eyebrow end (LBE).

**Figure 3:**
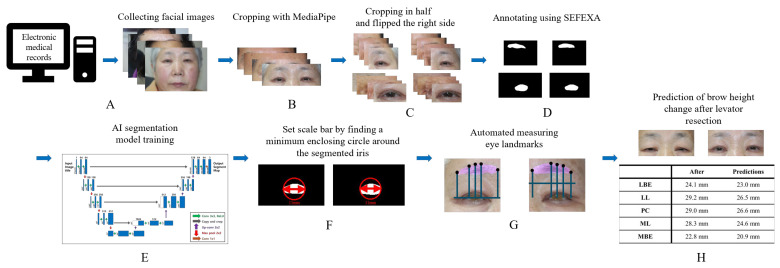
Entire processing strategy from data collection to data postprocessing. (**A**) Collecting facial images of 459 patients from electronic medical records. (**B**) Cropping the facial images around the iris and eyebrow, which was the region of interest. (**C**) Cropping the facial images in half to collect unilateral iris and eyebrow images. The right-side facial image was flipped to align the two facial images. (**D**) Annotating the unilateral images using the Sefexa tool to produce a dataset for developing the iris and eyebrow segmentation models. (**E**) Training segmentation models using U-Net as the architecture. (**F**) Measurements were standardized using the iris diameter as a 11 mm reference. Since the iris was partially covered by the eyelids, a minimum enclosing circle was drawn around the segmented iris to estimate the diameter. (**G**) The heights of key eye landmarks from the PC to the highest eyebrow position. (**H**) These measurements may aid future predictions or visualizations of eyebrow changes after ptosis surgery. AI, artificial intelligence.

To apply the iris and eyebrow segmentation models effectively, we measured key eye landmarks as shown in Figure 2: margin reflex distance 1 (MRD1), margin reflex distance 2 (MRD2), medial eyebrow end (MBE), medial limbus (ML), pupil center (PC), lateral limbus (LL), and lateral eyebrow end (LBE). To ensure measurement consistency, all values were standardized using a horizontal corneal diameter of 11 mm, based on the findings of Khng et al. [15]. The entire workflow from data collection to postprocessing is illustrated in Figure 3.

### 2.3. Dataset Composition: Iris and Eyebrow Segmentation Models

The deep-learning models for iris and eyebrow segmentation were developed using 612 image samples from 209 patients (81 males, 128 females; mean age 71.4 ± 12.2 years). The dataset images were randomly divided into three subsets: 80% (490 image samples) for training, 10% (61 image samples) for validation, and 10% (61 image samples) for testing. This distribution ensured robust model training and evaluations for both segmentation tasks.

### 2.4. Construction of Deep-Learning Models

The deep-learning model was built within the U-Net architecture with an EfficientNet-B0 encoder pretrained on ImageNet, implemented in PyTorch (v.2.3.1). The model received 3-channel RGB inputs resized to 64 × 64 pixels. The input size was chosen to ensure efficient training and inference while preserving sufficient detail within the cropped periocular region. The decoder used transpose convolutions with skip connections, and the final output layer generated a single-channel probability map with sigmoid activation.

The model was trained using a batch size of 16 for 1000 epochs, with an initial learning rate of 0.005. The learning rate scheduler reduced the rate by half if the validation loss did not improve for 10 consecutive epochs. For optimization, we used the Adam optimizer and the BCEWithLogitsLoss, which provides stable convergence for the binary segmentation tasks. Training was conducted on an NVIDIA GeForce RTX 3080 GPU (10 GB memory; NVIDIA Corp., Santa Clara, CA, USA) environment. The model performance was evaluated using accuracy, precision, recall, F1-score, and IoU.

### 2.5. Model Evaluation

#### 2.5.1. Iris and Eyebrow Segmentation Models Bulleted Lists Look Like

True-positive (TP), false-positive (FP), true-negative (TN), and false-negative (FN) values were used to evaluate the performances of the AI-based models for the following metrics: (1) accuracy, (2) precision, (3) sensitivity, (4) F1 score, and (5) intersection over union (IoU). Accuracy refers to the proportion of correctly predicted samples relative to the total number of samples; that is, (TP + TN)/(TP + TN + FP + FN). Precision is the ratio of TP samples to the total number of samples predicted as positive; that is, TP/(TP + FP). Sensitivity is the proportion of predicted positive samples among the TP samples; that is, TP/(TP + FN). There is a trade-off between precision and sensitivity, since as one increases the other tends to decrease.

The F1 score is a metric used to evaluate the performance of machine-learning models, particularly in classification tasks. It provides a balanced measure of the precision and sensitivity of a model, and ranges from 0 to 1, with higher values indicating better overall performance. The F1 score is calculated as 2 × (precision × sensitivity)/(precision + sensitivity). The IoU measures the degree of overlap between the ground truth and the predicted segmentation, relative to their total combined area; that is, TP/(TP + FP + FN). It quantifies how well the predicted segmentation aligns with the actual ground truth, with values ranging from 0 to 1, where higher values indicate higher accuracy.

#### 2.5.2. Quantitative Evaluation of Eye Landmarks

The eye landmark measurements were evaluated using the mean absolute percentage error (MAPE) and root mean square error (RMSE). Both of these metrics represent the prediction accuracy of the model, with values closer to 0 indicating better performance. MAPE expresses the difference between predicted and actual values as a percentage, and it is calculated as (100/n) × Σ|actual − predicted|/|actual|. RMSE expresses the error in the same units as the original data, and it is calculated as √(Σ(actual − predicted)^2^/n).

## 3. Results

The 612 image samples utilized in this study came from 209 patients with acquired ptosis aged 71.4 ± 12.2 years (mean ± SD), comprising 81 males and 128 females.

### 3.1. Iris and Eyebrow Segmentation Model

The performance characteristics of the iris and eyebrow segmentation models were evaluated using multiple metrics. The iris segmentation model achieved an accuracy of 99.7%, precision of 97.6%, recall of 98.3%, F1 score of 97.9%, and an IoU of 95.9%; the similarly good performance parameter values for the eyebrow segmentation model were 98.6%, 92.6%, 91.5%, 91.5%, and 85.0%, respectively. These results indicate that both models effectively performed their respective segmentation tasks, with the iris segmentation model exhibiting slightly better performance across all metrics. Notably, the superior performance of the iris model can be attributed to the clear contrast between the iris and sclera, whereas eyebrow segmentation showed slightly lower consistency due to natural variations in hair density and shape. Segmentation errors were most frequently observed in cases with sever ptosis or indistinct eyebrow margins, suggesting that the models are sensitive to anatomical variability.

### 3.2. Eye Landmark Measurements

The RMSE and MAPE values for seven key eye landmarks were as follows: MRD1 (0.261 mm and 10.7%), MRD2 (0.283 mm and 6.26%), MBE (4.42 mm and 1.78%), ML (4.66 mm and 1.85%), PC (2.22 mm and 1.81%), LL (2.75 mm and 2.81%), and LBE (2.75 mm and 2.79%). The overall RMSE and average MAPE across all eye landmarks were 2.48 mm and 4.00%, respectively, as summarized in Table 1.

Although MRD1 and MRD2 are clinically important parameters for evaluating eyelid position, the MAPE values were 10.7% and 6.26%, respectively. However, these percentages reflect smaller denominators in ptotic eyes rather than large absolute errors. Their mean absolute errors were below 0.5 mm, which is within commonly reported clinical repeatability for MRD measurements [16,17]. For MRD1, the relatively higher MAPE is expected given its smaller mean value of 2.44 mm compared with MRD2 of 3.54 mm.

By relative error, the central landmarks (PC, MBE, and ML) showed lower values, whereas the outer landmarks (LL and LBE) exhibited higher values, which reflects the greater anatomical variability in the lateral periocular region. Similarly, Peterson et al. reported that central landmarks demonstrated smaller relative errors, while outer landmarks showed larger errors [18]. Nevertheless, the average MAPE of five landmarks remained below 3%, indicating that the relative measurement accuracy was well maintained.

### 3.3. Model Inference Latency

We evaluated the model-only latency evaluation on 100 randomly sampled images using a CPU-only environment (Intel Core i5-8250U, Intel Corp., Santa Clara, CA, USA; 8 threads). The iris segmentation model showed an average latency of 73 ms (median 71 ms, p90 79 ms). The brow segmentation model showed an average latency of 79 ms (median 75 ms, p90 95 ms), corresponding to near-real-time performance suitable for clinical workflows.

## 4. Discussion

In this study, we developed a deep-learning framework for the automated segmentation of the iris and eyebrow, enabling precise periocular landmark measurements in patients with acquired ptosis. Our motivation was the clear clinical need for objective and standardized tools for periocular measurement. By replacing manual annotation with standardized, less operator-dependent quantification, the framework provides reproducible metrics that can document pre- and postoperative changes. These objective measurements may contribute to surgical planning and patient counseling.

The models achieved high metrics for iris segmentation and brow segmentation, which are accuracies of 99.7% and 98.6%, with an F1-score of 97.9% and 91.5% and IoU values of 95.9% and 85.0%, respectively. Automated landmark measurements—including MRD1, MRD2, medial brow end (MBE), medial limbus (ML), pupil center (PC), lateral limbus (LL), and lateral brow end (LBE)—further demonstrated an average MAPE of 4.00% and an RMSE of 2.48 mm. These metrics support integration as a decision-support tool with human-in-the-loop review in routine periocular practice.

**Figure 4:**
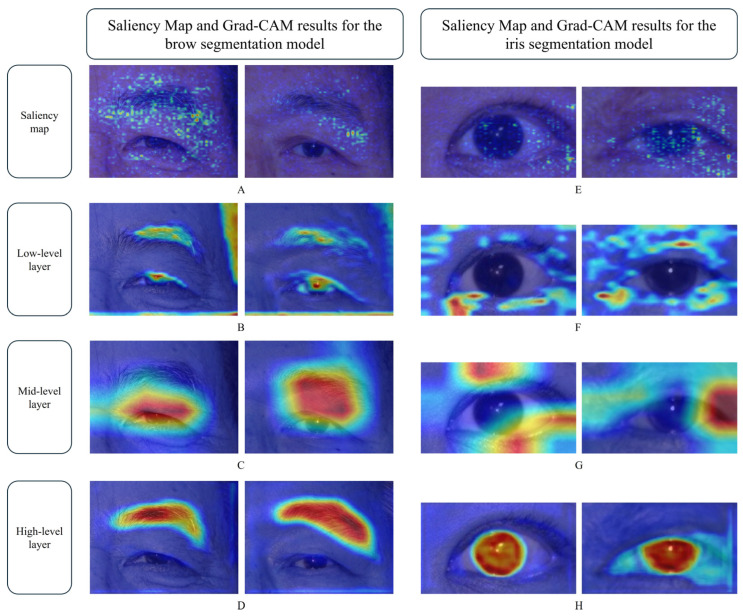
Visual interpretation of the model focuses using Grad-CAM and saliency maps for eyebrow (**A**–**D**) and iris (**E**–**H**) segmentation (Red/yellow areas indicate stronger activation). In the eyebrow segmentation model, (**A**) shows that the model relied on both the eyebrow and surrounding skin structures; (**B**) highlights the focus on fine edges such as the eyebrow, lashes, and hairline; (**C**) illustrates the focus on shading and texture below the eyebrow; and (**D**) reveals precise activation within the eyebrow area, reflecting the effective integration of spatial and semantic features. In the iris segmentation model, (**E**) shows the focus on both iris boundaries and adjacent skin features; (**F**) displays strong activation along the iris edge and nearby anatomical landmarks; (**G**) demonstrates the reliance on broader contextual cues such as skin texture and lighting characteristics; and (**H**) shows concentrated activation within the iris region, independent of the degree of iris visibility.

While our segmentation models exhibited high overall accuracies, we also conducted error analysis using explainable AI (XAI) techniques—specifically, saliency maps and Grad-CAM—to better understand the sources of segmentation errors and the decision-making process of the model (Figure 4). Saliency maps highlight the image regions that most influence the model output, offering insight into which features the model prioritized. Grad-CAM further visualizes class-specific activations by generating heat maps that indicate the regions most relevant to the model predictions.

**Figure 5:**
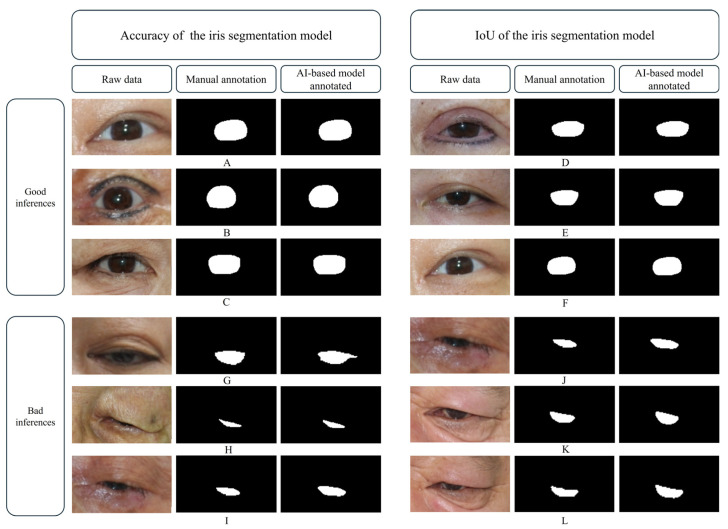
Results of the AI-based iris segmentation model compared with those of manual iris segmentation. (**A**–**C**) show high accuracy while (**D**–**F**) show high intersection over union (IoU) values. (**G**–**I**) show low accuracy while (**J**–**L**) show low IoU values. Patients with severe ptosis generally exhibited higher IoU values and accuracy.

During iris segmentation, the images with lower accuracy and IoU values frequently involved cases of severe ptosis, where the eyelid partially covered the iris boundary (Figure 5). The restricted visibility of the iris in such cases hindered the ability of the model to accurately delineate its contours. Additionally, misclassifications were observed in some cases where eyelashes were incorrectly included as part of the iris (Figure 5G).

To further examine these problems, we applied XAI methods to both the high- and low-performing cases. Saliency maps revealed that the model relied not only on the iris itself but also on surrounding anatomical cues such as eyelid folds, pupil margins, and skin texture. Grad-CAM results aligned with this observation: while earlier network layers responded broadly to iris-adjacent features, later layers showed more-concentrated activation within the iris region. These findings suggest that although the model integrated contextual information to compensate for obscured features, accurate segmentation was ultimately dependent on the degree to which the iris is visible. This explains the observed performance decline in cases of severe ptosis.

Errors during eyebrow segmentation were primarily attributed to interindividual variations in eyebrow shape and density [19]. Cases with sparse eyebrows or irregular eyebrow contours resulted in slightly lower segmentation accuracy. Notably, the AI-based model produced rounder and smoother segmentation contours relative to manual annotations, which may be attributed to pixel-level precision differences between model-based and manual annotation [20]. Employing a deep convolutional neural network fine-tuned using generative adversarial networks for annotation refinement could potentially overcome the limitations of the current dataset to improve segmentation accuracy [21]. To investigate this problem further, we applied saliency maps and Grad-CAM. The saliency maps showed that the model not only focused on the eyebrow itself but also relied heavily on surrounding contextual information, such as the eyelids and nearby skin texture. Grad-CAM further revealed that low-level decoder layers were activated in response to edge features such as eyebrows, eyelashes, and hairlines, while for mid-level layers the focus shifted to areas below the eyebrow. However, high-level decoder layers demonstrated distinct and localized activation within the eyebrow region. These results indicate that U-Net skip connections enabled the model to incorporate both spatial and semantic features, ultimately allowing it to focus accurately on the eyebrow despite shape and density variations.

**Figure 6:**
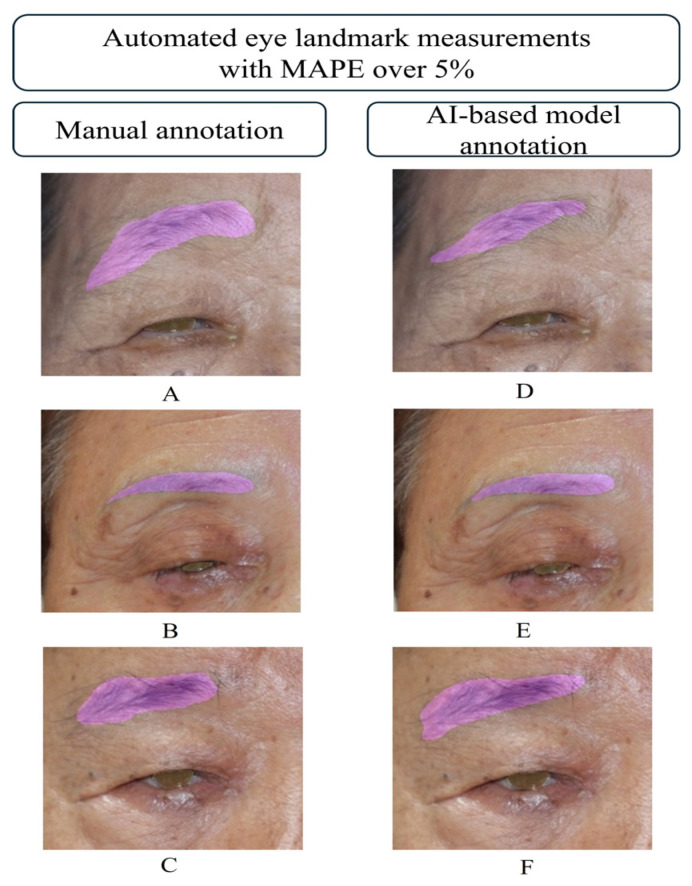
Examples of AI-based eyebrow segmentation model with a mean absolute error (MAPE) over 5% compared to manual annotations. (**A**–**C**) shows the manual annotation, and (**D**–**F**) show the corresponding AI-based segmentation results. These three examples represent the highest-error cases among the 11 out of 612 image samples where the MAPE exceeded 5%.

In the eye landmark measurements, the MAPE for MRD1 (10.7%) and MRD2 (6.26%) were higher than the average MAPE of 2.21% for the rest five eye landmarks. These discrepancies were probably due to differences at the small-scale level. In our dataset, the average MRD1 was 2.44 mm and the average MRD2 was 3.54 mm, which were smaller than the average for the five landmarks (55.4 mm), meaning that even slight measurement errors could result in large error metrics. The eye landmark measurements for the other five landmarks (MBE, ML, PC, LL, and LBE) all achieved average MAPE values below 3%. Only 11 of the 612 image samples showed MAPE values above 5% for these five landmarks, some of which are shown in Figure 6. Comparing segmentation results revealed no significant visible differences in iris segmentation, while some discrepancies were noted in eyebrow segmentation. The AI-based model annotations were either larger or smaller than those of the observers, leading to errors. Additionally, data points with large errors consistently showed high MRD1 errors, since cornea diameter which is directly related to the MRD1 was used for the scale bar.

The key contribution of this work lies in addressing the specific clinical context of acquired ptosis in East Asian patients. A major novelty of our study is that the segmentation models were trained and validated using a ptosis-specific dataset that includes both pre- and postoperative images, an approach that, to our knowledge, has been rarely reported. This clinically relevant dataset enhances the applicability of the model in real surgical settings. Compared with recent state-of-the-art segmentation approaches, our framework achieves comparable accuracy while extending beyond segmentation to provide clinically interpretable landmark measurements tailored to ptosis surgery. This positioning highlights the added value of our work in bridging technical performance with clinical utility.

Our iris segmentation model exhibited excellent performance, which are comparable to the values obtained for previous models of iris segmentation. For instance, the SAM-Iris model achieved high performance on benchmark datasets, with F1-scores, IoU, and accuracy of 95.15, 90.88, and 96.49 on CASIA.v4-Distance and 94.08, 88.94, and 94.97 on UBIRIS.v2, respectively [22]. Chen et al. developed an end-to-end unified framework integrating MADNet and DSANet (without normalization), reporting F1-scores of 97.40, 94.86, and 98.69 on ND-IRIS-0405, CA4D, and IITD datasets [23]. Similarly, Lin et al. proposed a domain-invariant segmentation framework that achieved F1 and IoU of 93.24 and 87.94 on UBIRIS.v2, 96.83 and 94.28 on ND-IRIS-0405, and 94.30 and 89.36 on IITD [24]. Compared with these works, our model shows comparable segmentation accuracy while being optimized for clinical periocular analysis rather than purely biometric tasks. Unlike public iris datasets which primarily comprise images of healthy subjects acquired under near-infrared illumination in well-controlled environments, our dataset consists of acquired ptosis cases captured under visible-light conditions. This setting better reflects real clinical acquisition while preserving standardized gaze and head position for ptosis severity documentation. Therefore, the commonly used public datasets may fail to reflect such real-world clinical variability. From this perspective, our dataset provides an advantage by representing ptosis-specific and practically relevant imaging conditions for iris segmentation while maintaining comparable quantitative performance.

Our brow segmentation model achieved good performance, with an accuracy of 98.6%, F1-score of 91.5%, and IoU of 85.0%, which are comparable to previous studies that segmented broader periocular regions rather than the eyebrow alone. For instance, Nahass et al. utilized CFD and CelebA datasets to segment periocular structures including sclera, iris, caruncle, lid, and brow, achieving a brow Dice score of 0.90 on CFD [25]. Similarly, Zeng et al. employed the HELEN and CelebAMask-HQ datasets and FP-Deeplab framework to segment multiple facial components such as the mouth, nose, and brow, reporting a brow F1-score of 84.2% [26]. Unlike these studies based on large public datasets, our dataset was specifically collected from elderly East Asian individuals with clinical features. While public datasets are valuable for model generalization, their limited representation of periocular diversity restricts applicability to elderly East Asian patients encountered in actual clinical practice. From this perspective, our study provides a clinically relevant dataset and benchmark performance that better represent actual patient populations. Moreover, Glass et al. reported that the lateral tail of the brow descends with age, with rates of change varying by sex and ethnicity [27]. Park et al. also demonstrated that in females, the lateral end point (EP) was significantly lower in older groups than in younger ones [28]. These findings underscore the age- and ethnicity-related variations in brow morphology, suggesting that models trained on public datasets may require validation for their performance in elderly East Asian populations.

Our automated landmark measurements demonstrated high accuracy, with RMSE values of 0.261 mm for MRD1 and 0.283 mm for MRD2. Across seven periocular landmarks, the overall mean absolute percentage error (MAPE) was 4.00%, and the average RMSE was 2.48 mm, comparable to previous studies. For instance, Cao et al. segmented medial and lateral area of the eyelid and cornea using a deep learning model, reporting biases of 0.04 mm and 0.66 mm for MRD1 and MRD2, respectively [17]. Similarly, Lou et al. compared manual and automated measurements of MRD1 and MRD2, yielding biases between 0.09 mm and 0.15 mm [16]. van Brummen et al. developed periorbitAI, a framework that segments iris, sclera, and eyebrow to automatically compute periorbital metrics such as MRD1, MRD2, MCH, and LCH [29]. While these studies successfully automated periorbital measurements, they did not evaluate eyebrow position, which can significantly change in ptosis patients and after blepharoplasty. Therefore, unlike prior frameworks, our study includes brow landmarks, addressing a key variable in ptosis and blepharoplasty.

A critical consideration for clinical translation is whether measurement error for MRD1 and MRD2 falls within the clinically accepted threshold. Previous studies have reported inter- and intra-observer repeatability of approximately ±0.5 mm for MRD measurements [16,17]. In our study, automated measurements of MRD1 and MRD2 achieved RMSE of 0.261 mm and 0.283 mm, respectively, indicating that the system’s performance is within this clinically meaningful range.

This study has several limitations. First, the dataset was derived from a single institution and predominantly consisted of East Asian elderly patients with involutional ptosis. While this reflects the typical clinical population for this condition, it may limit the generalizability of the model. However, we consider the effect of skin tone or age to be relatively minor, since eyebrows are usually darker than surrounding skin and eyelid margins are contrasted against the sclera, allowing for robust segmentation. Furthermore, XAI results (Figure 4) demonstrated that the model relied on common anatomical cues and skin texture, which are independent of skin tone or age. Nevertheless, additional validation across different ethnicities, age groups, eyelid morphologies, and imaging conditions is warranted. Future work should therefore include multicenter, cross-device validation to confirm robustness and clinical applicability. Second, a single annotator generated the labels. Future work should include multiple annotators and assess inter-annotator agreement (e.g., IoU, boundary distance) to enhance reliability. Third, cornea diameter was standardized to 11 mm for calibration, as this value lies within the typical adult range of 11–12.5 mm. While this assumption enabled consistent scaling of landmark measurements, it may not hold true for all individuals and could introduce measurement bias in certain cases. Future studies should validate this assumption using direct ocular measurements and analyze calibration sensitivity. Fourth, our preprocessing strategy relied on unilateral cropping and horizontal flipping to align right and left facial images. Although these approaches reduced irrelevant background and ensured consistent orientation for efficient training, it may also have limited the contextual information available to the model. Evaluations with larger periocular crops or full-face images without flipping could improve robustness, especially when eyebrow or eyelid boundaries are ambiguous. Future studies should quantitatively assess the effects of cropping extent and flipping on model performance to validate these preprocessing choices. Fifth, the model also showed sensitivity to domain shifts, particularly in severe ptosis, where iris occlusion reduced segmentation accuracy. Quantifying performance by severity and occlusion extent is needed.

Building upon these findings and acknowledging the model’s current limitations, a valuable next step would be to compare its performance with recent state-of-the-art architectures such as Attention U-Net, TransUNet, or Swin-Transformer–based networks, which have demonstrated superior performance in medical image analysis [10,11,12]. While the U-Net architecture achieved high segmentation accuracy in our study, benchmarking against these advanced architectures could provide deeper insights into performance and robustness, particularly in challenging cases such as severe ptosis or poorly defined eyebrow boundaries. Beyond technical performance, the clinical value of the proposed framework lies in its ability to link periocular landmark measurements to surgical outcomes. Therefore, adopting a human-in-the-loop approach where automated measurements serve as decision support tools and low confidence outputs are flagged for clinical review will be essential for clinical safety and adaptation. Furthermore, our analysis relied on pre- and postoperative images, without accounting for longitudinal changes in eyebrow and eyelid position that may occur during the healing process. Future studies should include temporal information by incorporating longitudinal follow-up data. This would allow the framework to capture the dynamic course of postoperative changes and provide more reliable predictions for clinical practice.

## 5. Conclusions

This study has produced a novel AI-based framework for iris and eyebrow segmentation along with automated eye landmark measurements in East Asian patients with acquired ptosis. The high accuracy and consistency of the proposed models demonstrate their potential for supporting objective and standardized clinical assessments. By reducing the subjectivity and inefficiency associated with manual annotation, this approach may increase reproducibility in periocular measurements and contribute to improved preoperative planning. These findings could ultimately form the groundwork for the future development of predictive tools to assist in surgical decision-making and patient counseling, particularly in anticipating the postoperative eyebrow position following ptosis correction surgery.

## Figures and Tables

**Table 1:** Values of the performance metrics RMSE and MAPE for automated measurements of eye landmarks based on AI-based segmentation models relative to those based on manual annotation.

Eye Landmarks	RMSE (mm)	MAPE (%)
MRD1	0.261	10.7
MRD2	0.283	6.26
MBE	4.42	1.78
ML	4.66	1.85
PC	2.22	1.81
LL	2.75	2.81
LBE	2.75	2.79

MBE, medial brow end; ML, medial limbus; PC, pupil center; LL, lateral limbus; LBE, lateral brow end.

## Data Availability

The data presented in this study are available upon request from the corresponding author due to restrictions imposed by the Institutional Review Board, which approved the study protocol.

## Abbreviations

The following abbreviations are used in this manuscript:

RMSE	Root mean squared error
MAPE	Mean absolute percentage error
MRD	Marginal reflex distance
MBE	Medial brow end
ML	Medial limbus
PC	Pupil center
LL	Lateral limbus
LBE	Lateral brow end
